# Whole exome sequencing enhances diagnosis of hereditary bronchiectasis

**DOI:** 10.1186/s13023-025-03661-z

**Published:** 2025-03-24

**Authors:** Wangji Zhou, Yixuan Li, Haixia Zheng, Miao He, Miaoyan Zhang, Qiaoling Chen, Christopher Situ, Yaqi Wang, Ting Zhang, Keqi Chen, Jinrong Dai, Shuzhen Meng, Xueqi Liu, Aohua Wu, Yaping Liu, Kai-Feng Xu, Xinlun Tian, Xue Zhang

**Affiliations:** 1 Department of Pulmonary and Critical Care Medicine, State Key Laboratory of Complex Severe and Rare Diseases, Peking Union Medical College Hospital, Chinese Academy of Medical Sciences, Peking Union Medical College, Beijing, China; 2 State Key Laboratory of Common Mechanism Research of Major Diseases, Peking Union Medical College Hospital, Chinese Academy of Medical Sciences, Peking Union Medical College, Beijing, China; 3 State Key Laboratory for Complex, Severe, and Rare Diseases, State Key Sci-tech Infrastructure for Translational Medicine, Peking Union Medical College Hospital, Beijing, 100730 China; 4 State Key Laboratory of Medical Molecular Biology, Institute of Basic Medical Sciences, McKusick-Zhang Center for Genetic Medicine, Chinese Academy of Medical Sciences & Peking Union Medical College, Beijing, China; 5 Department of Laboratory Medicine and Pathobiology, Faculty of Medicine, University of Toronto, Toronto, Canada

**Keywords:** Hereditary bronchiectasis, Whole exome sequencing, Diagnosis, Re-analysis

## Abstract

**Background:**

Hereditary bronchiectasis refers to a subset of bronchiectasis related to genetic mutations, presenting with common clinical features. Historically, diagnosing this condition has been difficult due to the inaccessibility of diagnostic services coupled with a lack of awareness of the syndrome. We hypothesize that whole exome sequencing (WES) in patients with supporting clinical features, combined with non-genetic testing methods, will enhance the diagnosis of hereditary bronchiectasis.

**Results:**

In total, 87 patients with clinical features suggestive of hereditary bronchiectasis, such as diffuse bronchiectasis (≥ 2 lobes) combined with early onset symptoms, recurrent otitis media, rhinosinusitis, infertility, organ laterality defects or a family history of bronchiectasis, were included in this study. Among them, 49.4% (43/87) were diagnosed with hereditary bronchiectasis, including 15 patients with cystic fibrosis, 27 patients with primary ciliary dyskinesia, and 1 patient with immunodeficiency-21. The combined use of WES and non-genetic testing methods significantly improved the diagnostic rate of hereditary bronchiectasis compared to non-genetic testing alone (47.1% vs. 25.3%, *P* = 0.005). Re-analysis of negative commercial genetic tests led to two additional diagnoses, though this increase was not statistically significant (47.1% vs. 49.4%, *P* = 0.879).

**Conclusions:**

We have described the supporting clinical features of patients with hereditary bronchiectasis. Clinicians should recommend WES for patients exhibiting these characteristics, in combination with accessible non-genetic testing methods, to maximize diagnostic accuracy. For patients with negative initial genetic test results, re-analysis of WES data may facilitate obtaining a new diagnosis.

**Supplementary Information:**

The online version contains supplementary material available at 10.1186/s13023-025-03661-z.

## Background

Bronchiectasis is a heterogeneous disease characterized by permanent enlargement of the airways [1]. It can result from infectious, genetic or immunological causes, but the cause of most cases remains unknown and is referred to as idiopathic. Hereditary bronchiectasis is a subset of the disease associated with genetic mutations, including conditions such as cystic fibrosis (CF), primary ciliary dyskinesia (PCD), alpha-1 antitrypsin deficiency, and immunodeficiency [2]. Guidelines recommend investigating the underlying causes of bronchiectasis due to their significant therapeutic and prognostic implications [3].

For various reasons, hereditary bronchiectasis remains an underdiagnosed category of bronchiectasis. Firstly, in the United States and some European countries, CF newborn screening such as blood trypsinogen has been routinely conducted. For patients with elevated levels of blood trypsinogen, genetic testing for common *CFTR* variants will be performed. In these countries, the underdiagnosis of CF may be due to patients having sufficient pancreatic function or to the presence of uncommon *CFTR* variants in non-White populations [4]. Moreover, pancreatic exocrine insufficiency may not be manifested in the early age, leading to missed diagnosis. Clinical follow-up is also important. For infants who had a positive screening test for CF but received an inconclusive diagnosis, approximately 20% of them will subsequently meet diagnostic criteria for CF [4]. Secondly, the prevalence of hereditary bronchiectasis is relatively low. According to statistics, CF, PCD and immunodeficiency account for 0.6-2.7%, 0.9-10.3%, and 5% of adult bronchiectasis patients, respectively [3, 5]. Considering the rarity of CF in Asian populations, the prevalence may be even lower [6]. This limited prevalence may lead to insufficient awareness of these conditions among clinicians. Thirdly, patients with hereditary bronchiectasis exhibit common clinical characteristics that require careful identification for proper diagnosis. Most patients have symptoms originating in childhood and exhibit diffuse bronchiectasis (involving two or more lobes). Moreover, their clinical manifestations are similar, including a history of recurrent respiratory infections, neonatal distress, rhinosinusitis, and recurrent otitis media. Infertility is also common in both CF and PCD patients [7]. However, it is worth noting that although the above symptoms are common in patients with hereditary bronchiectasis, they are rare in patients with bronchiectasis caused by other reasons, which is an important clue for identifying patients with hereditary bronchiectasis [8]. Finally, diagnostic testing for hereditary bronchiectasis is complex and often inaccessible. Diagnosing CF involves sweat chloride testing and cystic fibrosis transmembrane conductance regulator (*CFTR*) mutation analysis [9]. According to guidelines set by the American Thoracic Society (ATS), diagnosing PCD requires genetic testing, nasal nitric oxide (nNO) measurement, and transmission electron microscopy (TEM) analysis [10]. Similarly, serum immunoglobulin testing and genetic testing may be necessary to identify bronchiectasis caused by immunodeficiency [3].

At present, the guidelines recommend *CFTR* sequencing (for CF) or gene-panel sequencing (for PCD) for hereditary bronchiectasis [9, 11]. However, due to the different *CFTR* gene profiles in different populations (such as the p. Gly970Asp mutation being more common in Chinese people than the p. Phe508del mutation being the most common in Caucasians) [12], the types of pathogenic genes in PCD are diverse and constantly increasing [13], and the causes of hereditary bronchiectasis are diverse and difficult to distinguish, which limit the use of the above genetic testing methods. Whole exome sequencing (WES) is a genomic technique that analyzes all protein-coding regions, known as exons, in a genome. Its ability to efficiently identify disease-causing genetic variants has revolutionized the diagnosis of monogenic disorders [14]. In addition to being relatively non-invasive and accessible, WES can distinguish between different etiologies of hereditary bronchiectasis and help identify novel disease-implicated mutations. However, the use of WES is limited by its cost and the inability of negative sequencing results to conclusively exclude a diagnosis [11]. Therefore, a combination of multiple diagnostic tests, supplemented with selective WES when appropriate, is required during diagnosis.

In this study, we performed WES on patients exhibiting clinical features of hereditary bronchiectasis and re-analyzed the raw data of those with negative results from commercial genetic tests. We also used non-genetic testing methods such as physical examinations, sweat chloride testing, nNO measurement, and TEM analysis to improve the diagnosis of hereditary bronchiectasis.

## Methods

### Subjects

All patients included in the study were recruited from the outpatient and inpatient departments of the Department of Pulmonary and Critical Care Medicine at Peking Union Medical College Hospital (PUMCH) between August 2022 and April 2024. The inclusion criteria were as follows: (1) Diagnosis of diffuse bronchiectasis by chest CT (≥ 2 lobes, with separate calculations for the left upper lobe and left lingual lobe); (2) Presence of at least one of the following supporting clinical features of hereditary bronchiectasis [3]: ① Childhood-onset symptoms; ② Rhinosinusitis or otitis media; ③ Organ laterality defects; ④ Reproductive dysfunction, such as a history of abortion, infertility, decreased sperm motility, assisted reproduction, etc.; ⑤ Family history of bronchiectasis; (3) Consent obtained for WES. Known non-hereditary cases of bronchiectasis were excluded based on clinical history and laboratory examinations, including bronchiectasis following tuberculosis infection, secondary immunodeficiency (e.g. due to prolonged use of immunosuppressive drugs or human immunodeficiency virus infection), airway obstruction, recurrent aspiration, or connective tissue diseases.

Informed consent was obtained from all patients or their legal guardians. The study was approved by the institutional review board (IRB) of PUMCH (I-24PJ0537) and conducted in accordance with the tenets of the Declaration of Helsinki.

### Clinical assessments

Patients were first evaluated for demographic information (including age, gender) and medical history (including time of onset, comorbidities, fertility history, family history). This was followed by physical examinations (with particular attention to organ laterality defects), chest CT scans, sweat chloride testing, nNO measurement, TEM analysis, and WES. While not all patients underwent sweat chloride testing, nNO measurement, or TEM analysis, all patients underwent WES.

Samples for WES were collected from peripheral blood and analyzed by a third-party commercial genetic testing company. According to guidelines from the American College of Medical Genetics and Genomics, the presence of biallelic autosomal recessive pathogenic or likely pathogenic variants, or a monoallelic X-linked (in males) or autosomal dominant pathogenic or likely pathogenic variant, constituted a positive result [15]. The costs associated with WES were afforded by the PUMCH Public Welfare Project for Rare Disease Service Improvement (UPWARDS).

### Sweat chloride testing

According to the third edition of the guidelines published by the Clinical and Laboratory Standards Institute, sweat was collected from both pre-cleaned upper limbs of the patients. The current was gradually set to 4 mA and maintained for 5 min, while 0.5% pilocarpine nitrate and 0.05 mmol/l magnesium sulfate were used in iontophoresis to stimulate sweat. Sterile gauze (5.1 × 5.1 cm), pretreated with deionized water and air dried, was covered by waterproof surgical tape and used to collect sweat for 30 min. We evaluated the amount of collected sweat by weight. Sweat [Na^−^], [Cl^−^] and [K^−^] were measured in triplicate using a chemistry analyser (A&T EA07 Electrolyte analyser, A&T Corporation, Japan). All of the patients were tested twice [16].

### nNO measurement

According to the ATS recommendation, nNO was measured during quiet exhalation using the Nano Coulomb Breath Analyzer (Sunvou-CA2122, Wuxi, China) in cooperative children > 5 years of age and adults [10]. nNO production (in nanoliters per min) was calculated by multiplying nNO concentration (parts per billion) by sampling flow rate (0.6 L/min).

### TEM analysis

Fresh bronchial mucosas were obtained by bronchial biopsy. The samples were immersed in a fixative solution (2.5% glutaraldehyde). After post fixation in osmium tetroxide for an hour, progressive dehydration with graded ethanol, and being embedded in Epon 812, the samples were sliced into 600 nm sections and stained with tolonium chloride to localize the target structure under light microscopy. Selected regional sample sections were stained with uranyl acetate and lead citrate and photographed via TEM with an acceleration voltage of 80kV [17].

### WES raw data re-analysis

For patients with negative genetic test results, two independent reviewers (W. Z. and Y. Li) used Franklin by Genoox (https://franklin.genoox.com) to re-analyze the raw data provided in variant call format (VCF) files by the commercial company. The analysis focused on genetic variants with a minor allele frequency below 0.01 in aggregated databases (1000 Genomes, ESP6500, ExAC, gnomAD, and UK10K), located within exons or classical splice sites. Synonymous mutations and variants classified as benign or likely benign by the Franklin system were excluded, alongside variants with failed sequencing quality. Disagreements were resolved through consensus among all authors. Though the investigation primarily focused on previously reported mutations associated with bronchiectasis, a comprehensive evaluation of variants across multiple genes was also conducted to identify potentially novel pathogenic genes responsible for hereditary bronchiectasis. Confirmed variants were subsequently verified by *Sanger* sequencing.

### Diagnostic criteria

According to current guidelines, CF diagnosis was warranted if a patient exhibited relevant clinical features or had a positive family history and met one of the following criteria: (1) Sweat chloride value ≥ 60 mmol/L; (2) Intermediate sweat chloride value (30–59 mmol/L) along with 2 CF-causing *CFTR* mutations [9].

PCD was diagnosed if at least one of the following criteria was met: (1) Low nNO levels (< 77nL/min, excluding CF) combined with at least two of the four key clinical features of PCD, namely, unexplained neonatal respiratory distress in a term infant, year-round daily cough beginning before 6 months of age, year-round daily nasal congestion beginning before 6 months of age, or organ laterality defects; (2) Ciliary ultrastructural defects identified by TEM analysis; (3) Biallelic pathogenic variants in PCD-associated genes; (4) Kartagener syndrome [10, 18].

### Statistical analysis

Data analysis was conducted using SPSS version 22.0 software (IBM SPSS, USA). Continuous variables were reported as mean ± standard deviation or median (interquartile range), while categorical variables were described as the proportional percentage, No. (%). The chi-squared test was used to compare categorical variables. *P* < 0.05 was deemed statistically significant for all analyses.

## Results

### Demographic and non-genetic testing results of patients

In total, 87 patients with supporting clinical features of hereditary bronchiectasis were included in this study. Among them, 32 (36.8%) were male and 55 (63.2%) were female. The average age was 29.8 ± 12.9 years (range, 8 to 69), and 71 (83.5%) of the patients were adults. The demographic information and clinical manifestations of all patients are detailed in the supplementary document (e-Table 1).

**Fig. 1:**
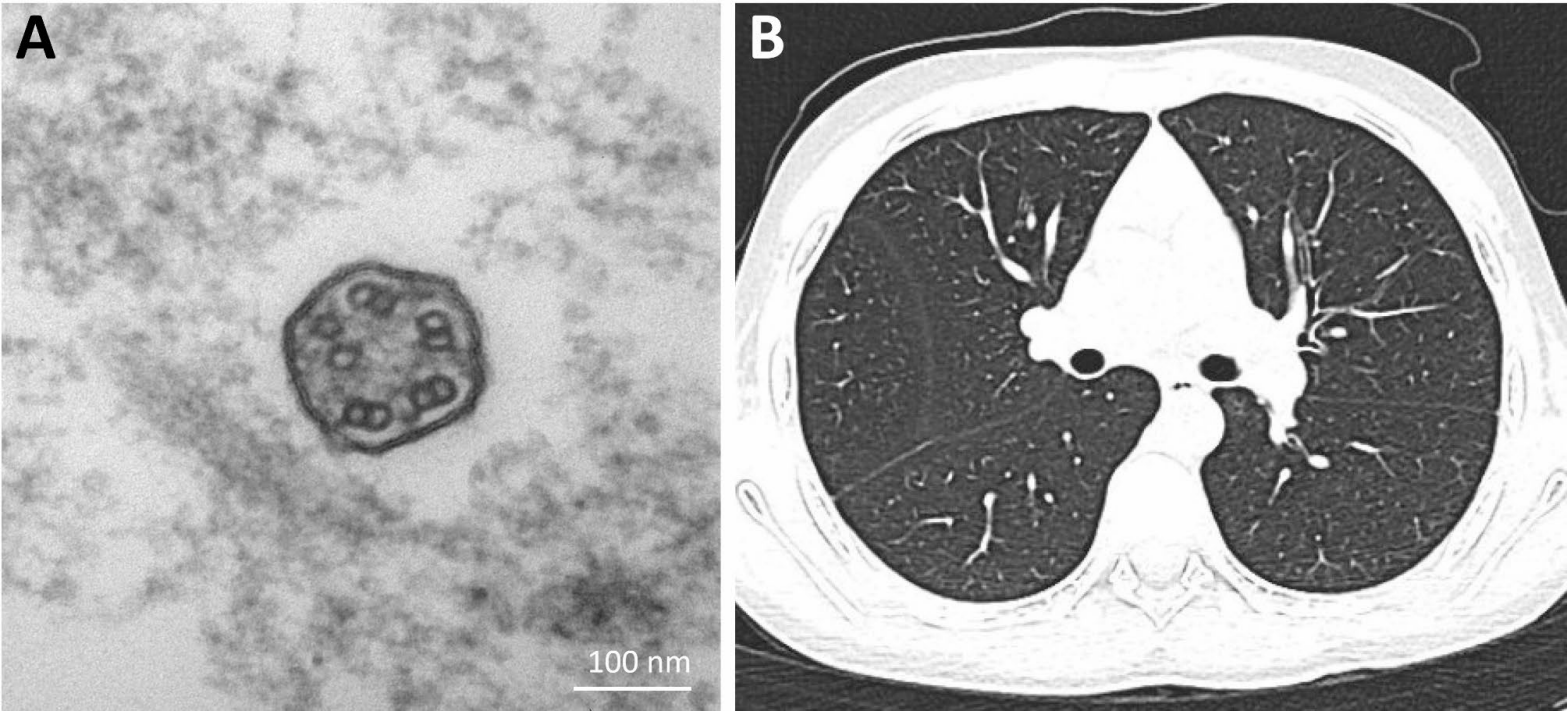
**A**: The TEM results of a PCD patient (No. 37 in e-Table 1) showed central pair absent and microtubular disorganization in bronchial cilia; **B**: Chest CT scan of a CF patient (No.7 in e-Table 1) indicates slight bronchiectasis in both lungs, especially in the upper lobes

Nineteen (21.8%) patients underwent sweat chloride testing, resulting in 10 positive diagnoses of CF. Eight (9.2%) patients had organ laterality defects combined with sinusitis and bronchiectasis, indicative of Kartagener syndrome. Four (4.6%) patients underwent TEM analysis of the bronchial mucosa, revealing two cases of ciliary ultrastructural defects characteristic of PCD. Figure 1A shows the TEM results of patient No. 37 in e-Table 1, who has central pair absent and microtubular disorganization and can be diagnosed with PCD according to the 2018 ATS guidelines. Among the 64 (73.6%) patients who underwent nNO measurement, about half (33/64, 51.6%) had low nNO levels. Two of these patients had at least two key clinical features for PCD, and subsequent WES, which did not detect *CFTR* mutations, confirmed the clinical diagnosis of PCD. The non-genetic testing results of all patients are detailed in e-Table 2.

### WES results reported by commercial genetic testing company

All 87 patients underwent WES, with 36 (41.4%) patients reporting positive results. The genes with mutations reported in multiple patients were *CFTR* (14 individuals), *DNAH5* (5 individuals), *DNAH11* (4 individuals) and *DNAAF11* (2 individuals). Additionally, mutations in *CCNO*, *CFAP300*, *DNAAF1*, *DNAAF4*, *DNAAF6*, *ODAD1*, *OFD1*, *RSPH3*, *RSPH4A*, *RSPH9* and *GATA2* were found in individual patients. Among these, *CFTR* is the pathogenic gene for CF, *GATA2* is associated with immunodeficiency-21, and the remaining 13 genes are associated with PCD. Specific information on patient genetic variation is detailed in the e-Table 2.

### Re-analysis of the Raw genetic testing data

After reanalyzing the raw data provided by the commercial company of 51 patients who initially reported negative results, missed diagnoses were confirmed in 2 patients. One patient is a 9-year-old male (patient No.7 in e-Table 1) who was born with intrahepatic cholestasis and occasionally has expectoration. Chest CT indicates slight bronchiectasis in both lungs, especially in the upper lobes (Fig. 1B). Sweat chloride testing is in the intermediate range (34/41 mmol/L). He carried two *CFTR* variants, c.3406G > A (p.Ala1136Thr) (PM2  + PP3) (PM refers to pathogenic moderate, PP refers to pathogenic supporting) and c.214G > A (p.Ala72Thr) (PM2  + PP3), which were originally reported as variants of uncertain significance (VUS). However, re-analysis classified both c.3406G > A (PM1 + PM2  + PP2 + PP3 + PP5) and c.214G > A (PM1 + PM2 + PM3 + PP2 + PP3) as likely pathogenic. Another patient is suspected to carry a novel PCD-causing gene, as the patient’s younger brother shares this gene variant and also has a PCD-related phenotype. Because the relevant research results have not yet been published, they are not detailed here.

### WES improves the diagnosis of hereditary bronchiectasis in patients with supporting clinical features

**Fig. 2:**
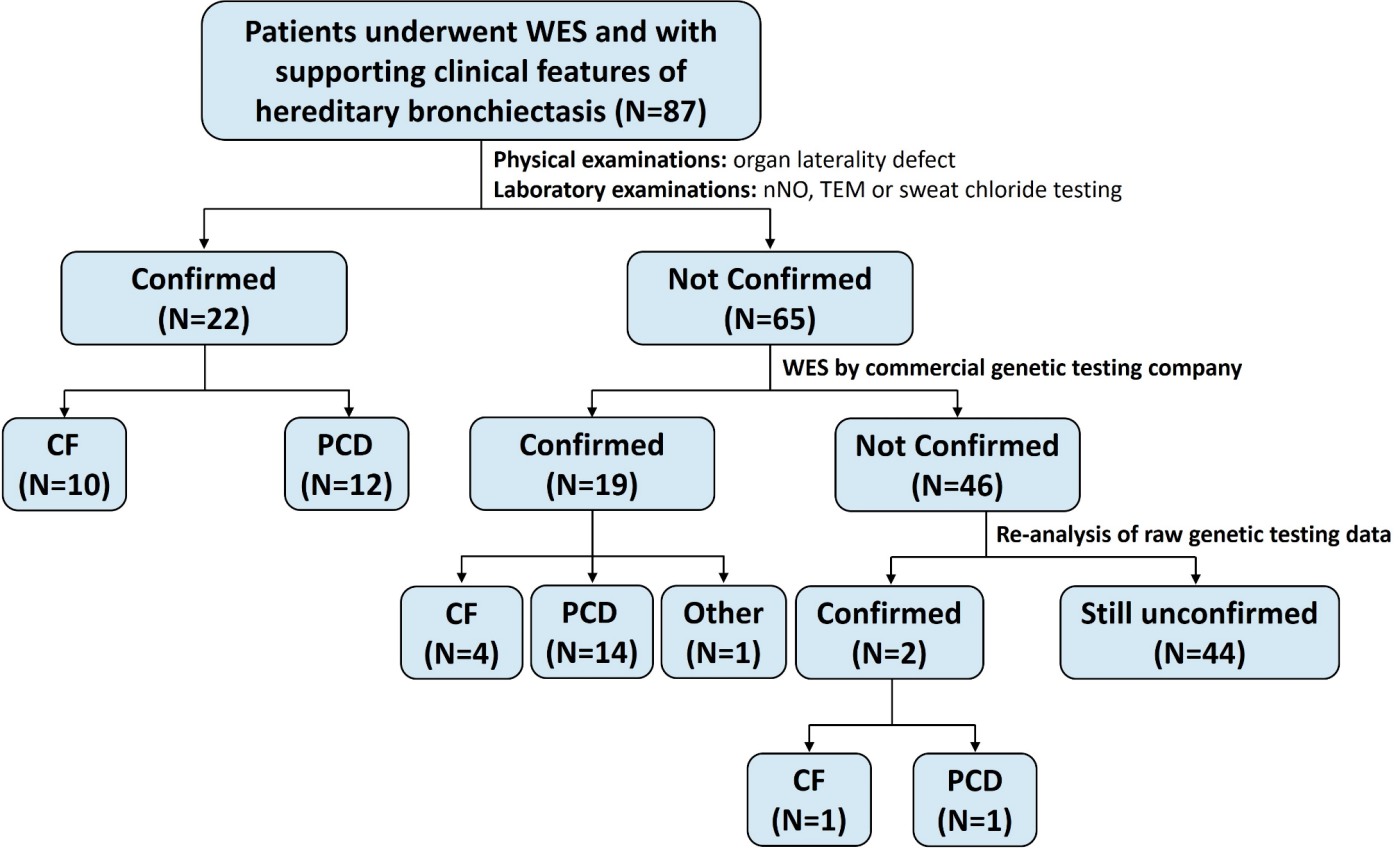
Diagnostic flow diagram for patients with supporting clinical features of hereditary bronchiectasis. WES: whole exome sequencing; nNO: nasal nitric oxide; TEM: transmission electron microscopy; CF: cystic fibrosis; PCD: primary ciliary dyskinesia

**Fig. 3:**
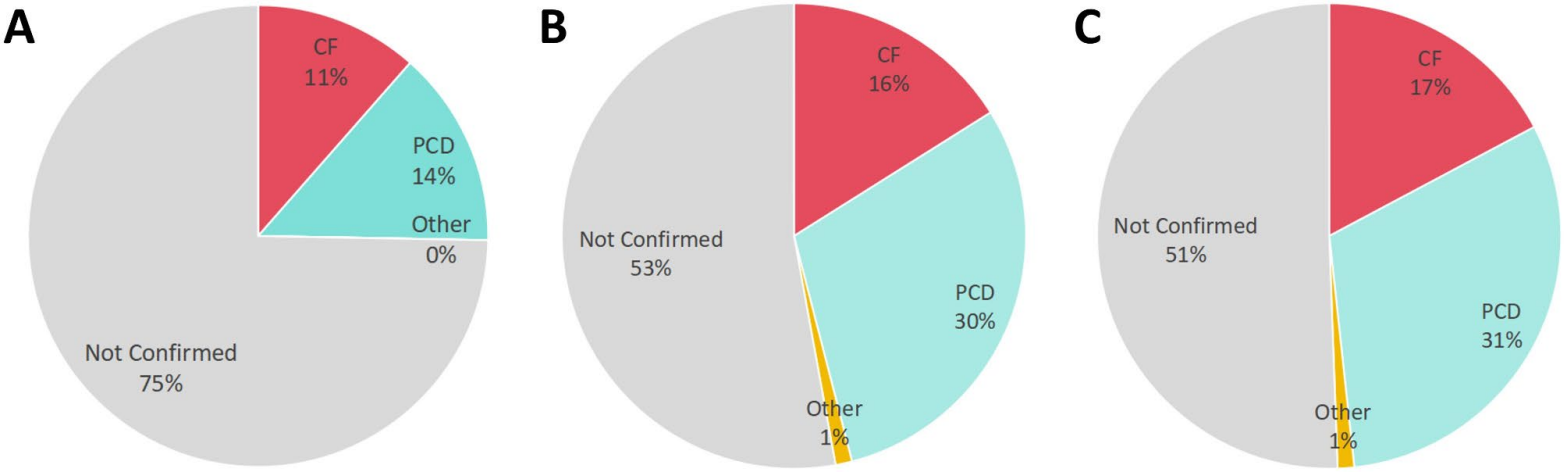
The proportion of patients diagnosed using different diagnostic methods. **A**: Non-genetic testing methods alone. **B**: Combining non-genetic testing methods with WES from a commercial genetic testing company. **C**: After re-analysis of raw genetic testing data as detailed above. CF: cystic fibrosis; PCD: primary ciliary dyskinesia

Among the original 87 patients with supporting clinical features of hereditary bronchiectasis, 22 (25.3%) were diagnosed through non-genetic testing methods. Following WES results from a commercial genetic testing company, diagnoses were confirmed for 19 of the remaining 65 patients. Thus, the combined diagnostic rate of non-genetic testing methods with WES was 47.1% (41/87). Re-analysis of raw genetic testing data provided clear diagnoses for two additional patients. In total, 43 patients (49.4%) were diagnosed with hereditary bronchiectasis, including 15 (17.2%) with CF, 27 (31.0%) with PCD, and 1 (1.2%) with immunodeficiency-21. The diagnostic flow diagram and results are depicted in Figs. 2 and 3.

Compared to using non-genetic testing methods alone, the combined use of WES significantly improved the diagnostic rate of patients with supporting clinical features of hereditary bronchiectasis (*P* = 0.005). However, re-analysis of the raw genetic testing data did not yield a significant improvement in the patient diagnostic rate (*P* = 0.879).

## Discussion

Our study conducted WES on 87 patients with supporting clinical features of hereditary bronchiectasis and re-analyzed the raw genetic testing data for patients who initially reported negative results by the commercial company. Compared with non-genetic testing methods alone, we found that utilizing WES significantly improved the diagnostic rate of hereditary bronchiectasis. Additionally, approximately half of the patients in this study were ultimately diagnosed with hereditary bronchiectasis, a proportion significantly higher than what is observed in general bronchiectasis patients. This suggests that hereditary bronchiectasis may manifest distinct supporting clinical features.

Hereditary bronchiectasis constitutes a small proportion of bronchiectasis cases, accounting for about 5–10% of adult patients with the condition [5]. According to a previous study, CF and PCD are relatively common among cases of hereditary bronchiectasis in China [19]. CF is one of the most common autosomal recessive diseases among Caucasians and is caused by mutations in the *CFTR* gene. However, CF is quite rare in China, with only about 200 reported cases to date [6]. The 2017 consensus guidelines for CF diagnosis from the Cystic Fibrosis Foundation recommend sweat chloride testing and *CFTR* genetic analysis to establish the diagnosis of CF. For individuals with sweat chloride values in the intermediate range (30–59 mmol/L), *CFTR* genetic analysis is necessary to confirm the diagnosis. Nevertheless, some *CFTR* mutations, such as c.3717 + 12,191 C > T, are associated with low sweat chloride values (< 30 mmol/L) [9], which can result in under-diagnosis of certain CF patient populations. PCD is a rare autosomal recessive or X-linked disorder caused by mutations in genes that encode the structure or function of motile cilia. As of May 2021, there were only 244 reported patients with PCD in China [20]. The ATS Guidelines recommend genetic testing, nNO measurement and TEM analysis for the diagnosis of PCD. However, it should be noted that some patients with PCD may have normal results in these tests, meaning while these tests can help confirm a PCD diagnosis, they cannot conclusively rule it out [11]. These challenges illustrate the difficulties in diagnosing hereditary bronchiectasis, which prompted our study to explore the following approaches.

Firstly, as the diagnostic methods for hereditary bronchiectasis are only available in a few centers, it is important to identify potential patients and refer them to specialized centers for definitive diagnosis. The British Thoracic Society Guideline recommends testing for CF in patients with supporting clinical features such as early onset symptoms and male infertility. Likewise, it recommends testing for PCD in patients with supporting clinical features such as childhood-onset symptoms, recurrent otitis media, rhinosinusitis, or infertility [3]. Building on this basis, our study included two additional criteria: organ laterality defects and a family history of bronchiectasis. The results showed that about half of the patients in this study were diagnosed with hereditary bronchiectasis, a proportion significantly higher than that in the general bronchiectasis population. Therefore, these criteria provide a valuable framework to help clinicians identify suspected patients with hereditary bronchiectasis. However, it should be noted that our study did not indicate the weight of these clinical features in suggesting the diagnosis of hereditary bronchiectasis, which requires further research.

Secondly, this study chose WES instead of gene-panel sequencing as the main method for diagnosing hereditary bronchiectasis. Gene-panel sequencing cannot identify novel genes, and panels designed for Caucasian populations may not be suitable for specific population, such as Chinese population [4, 12]. WES has the potential to revolutionize the diagnosis of monogenic disorders through massively parallel sequencing of almost all coding regions of the human genome, enabling simultaneous study of all known genes associated with genetic conditions. Previous studies have reported diagnostic rates ranging from 22 to 30% across heterogeneous indications [21]. Furthermore, in children suspected of having monogenic diseases, Tan et al. reported that WES is more cost-effective when used at initial presentation to tertiary care than the standard diagnostic pathway [14]. Other notable advantages of WES include its relative non-invasiveness, greater accessibility, ability to distinguish between different etiologies of hereditary bronchiectasis, and capacity to identify novel mutations. In this study, 41.4% of patients were diagnosed with hereditary bronchiectasis through WES reports from commercial genetic testing companies, which is slightly higher than in previous studies. By combining WES with non-genetic testing methods, the diagnostic rate improved to 47.1%. Therefore, we recommend that patients with clinical features suggestive of hereditary bronchiectasis consider WES as a diagnostic method, along with other non-genetic testing methods.

Finally, we re-analyzed raw genetic sequencing data from patients initially reported as negative by commercial genetic testing companies. Tan et al. summarized 27 studies that re-analyzed exome or genome sequencing data, revealing new diagnosis rates ranging from 0.08 to 83.34% (median 15%, weighted average 7%) [22]. The most successful strategies highlight several important approaches, including re-evaluating variants through segregation or functional analysis for reclassification, reanalyzing data using enhanced bioinformatic pipelines, and exploring new disease-gene associations [23]. In this study, two patients (3.9%) with initially negative genetic test results were subsequently diagnosed through VUS reclassification and the discovery of a novel pathogenic gene, respectively. This suggests that for patients with negative test results, re-analysis of the raw sequencing data can lead to novel diagnoses. In clinical practice, we recommend re-analysis for patients with high clinical suspicion of hereditary bronchiectasis, such as those with multiple supportive clinical features, decreased nNO levels, or abnormal sweat chloride levels. This will reduce the number of patients requiring reanalysis and increase the positivity rate.

This study has several limitations. Firstly, there is selection bias in this study. Since PUMCH is the largest rare disease diagnosis and treatment center in China, there is likely a disproportionately high representation of patients with hereditary bronchiectasis. Nevertheless, we believe that the inclusion criteria used to narrow down the patient population can be extended to other hospitals not specializing in rare diseases. Secondly, due to limited diagnostic resources, financial considerations, time constraints, and patients’ concerns about the discomfort of awake fiberoptic bronchoscopy, not all patients have undergone all non-genetic testing, which may overstate the diagnostic value of WES to a certain extent. Thirdly, our re-analysis utilized VCF files. Although VCF files provide valuable information on variant position and type, their functional annotation of variants, such as their impact on protein function and pathogenicity, relies on external databases and tools, which may result in incomplete or erroneous annotations [24]. Fourthly, some variants, such as certain structural variants (e.g., copy-neutral inversions; small, largely intronic copy-number variants; or complex events involving multiple types of structural variants), tandem repeat expansions, and deep intronic variants, are difficult to detect through WES and may require whole genome sequencing or other emerging genetic testing technologies [25, 26]. Lastly, while the costs associated with WES were covered by UPWARD for patients in this study, the accessibility of this screening method is currently limited to those who can afford it. However, ongoing technological advancements are expected to reduce the cost of WES, increasing its viability as a screening method for future patients.

## Conclusions

We have described the supporting clinical characteristics of patients with hereditary bronchiectasis. Clinicians should recommend WES for patients exhibiting these characteristics, complementing accessible non-genetic testing methods to enhance diagnostic accuracy. For patients with negative genetic test results, re-analysis of WES data may be a useful diagnostic approach.

## Electronic supplementary material

Below is the link to the electronic supplementary material.

Supplementary Material 1

## Data Availability

The data described in this manuscript was collected by the research team during the study period and can be obtained from the corresponding authors upon reasonable request.
