# Supplementary material for: Whole exome sequencing enhances diagnosis of hereditary bronchiectasis
Source: Orphanet J Rare Dis. 2025 Mar 24;20:142. doi: 10.1186/s13023-025-03661-z (PMC11934690; doi:10.1186/s13023-025-03661-z)
Supplement: Supplementary file 1 — Supplementary Material 1 [file 13023_2025_3661_MOESM1_ESM.docx]

**e-Table 1** Demographics and clinical characteristics of all 87 patients

| **Patient No.^§^** | **Demographic information** | | **Clinical characteristics** | | | | | | **Diagnosis** |
| --- | --- | --- | --- | --- | --- | --- | --- | --- | --- |
|  | **Gender** | **Age**  **(years)** | **Respiratory symptoms from childhood** | **Rhinosinusitis** | **Otitis media** | **Organ laterality defects** | **Reproductive dysfunction** | **Family history of bronchiectasis** |  |
| 1 | F | 18 | Y | Y | N | N | NA | N | CF |
| 2 | M | 21 | Y | Y | N | N | Y | N | CF |
| 3 | F | 29 | Y | Y | N | N | NA | N | CF |
| 4 | M | 8 | Y | Y | N | N | NA | N | CF |
| 5 | F | 16 | Y | Y | N | N | NA | N | CF |
| 6 | F | 20 | Y | Y | N | N | NA | N | CF |
| 7 | M | 9 | Y | N | N | N | NA | N | CF |
| 8 | M | 29 | Y | Y | N | N | N | Y | CF |
| 9 | F | 24 | Y | Y | N | N | NA | N | CF |
| 10 | F | 23 | Y | N | N | N | NA | N | CF |
| 11 | F | 18 | Y | N | N | N | NA | N | CF |
| 12 | F | 27 | Y | N | N | N | NA | N | CF |
| 13 | F | 19 | Y | N | N | N | NA | N | CF |
| 14 | M | 35 | Y | N | Y | N | Y | N | CF |
| 15 | M | 22 | Y | Y | N | N | Y | N | CF |
| 16 | M | 28 | Y | Y | N | N | Y | N | PCD |
| 17 | M | 32 | Y | Y | Y | N | N | N | PCD |
| 18 | M | 32 | Y | Y | N | N | N | N | PCD |
| 19 | F | 17 | Y | Y | N | N | NA | N | PCD |
| 20 | F | 33 | Y | Y | Y | Y | N | N | PCD |
| 21 | M | 55 | Y | Y | N | Y | Y | N | PCD |
| 22 | F | 32 | Y | Y | N | N | Y | N | PCD |

(Continued)

**e-Table 1** *(Continued)*

| **Patient No.^§^** | **Demographic information** | | **Clinical characteristics** | | | | | | **Diagnosis** |
| --- | --- | --- | --- | --- | --- | --- | --- | --- | --- |
|  | **Gender** | **Age**  **(years)** | **Respiratory symptoms from childhood** | **Rhinosinusitis** | **Otitis media** | **Organ laterality defects** | **Reproductive dysfunction** | **Family history of bronchiectasis** |  |
| 23 | M | 34 | Y | Y | N | N | Y | N | PCD |
| 24 | F | 28 | Y | Y | N | N | Y | N | PCD |
| 25 | F | 30 | N | Y | N | Y | NA | N | PCD |
| 26 | F | 16 | Y | Y | Y | N | NA | N | PCD |
| 27 | M | 15 | Y | Y | N | N | N | N | PCD |
| 28 | F | 39 | Y | Y | N | N | N | N | PCD |
| 29 | F | 26 | Y | Y | Y | Y | NA | N | PCD |
| 30 | M | 16 | Y | Y | N | N | NA | N | PCD |
| 31 | M | 43 | Y | Y | N | Y | Y | N | PCD |
| 32 | M | 15 | Y | Y | N | Y | NA | N | PCD |
| 33 | F | 39 | Y | Y | Y | Y | NA | N | PCD |
| 34 | M | 37 | N | Y | N | Y | N | N | PCD |
| 35 | M | 26 | N | Y | N | N | Y | N | PCD |
| 36 | M | 19 | Y | Y | N | N | NA | N | PCD |
| 37 | F | 32 | Y | Y | Y | N | Y | Y | PCD |
| 38 | F | 38 | Y | Y | N | N | Y | N | PCD |
| 39 | M | 18 | Y | Y | N | N | Y | N | PCD |
| 40 | F | 14 | Y | N | N | N | NA | N | PCD |
| 41 | F | 21 | Y | Y | Y | N | NA | N | PCD |
| 42 | F | 34 | Y | Y | N | N | Y | Y | PCD |
| 43 | F | 16 | Y | Y | N | N | NA | N | Immunodeficiency-21 |

(Continued)

**e-Table 1** *(Continued)*

| **Patient No.^§^** | **Demographic information** | | **Clinical characteristics** | | | | | | **Diagnosis** |
| --- | --- | --- | --- | --- | --- | --- | --- | --- | --- |
|  | **Gender** | **Age**  **(years)** | **Respiratory symptoms from childhood** | **Rhinosinusitis** | **Otitis media** | **Organ laterality defects** | **Reproductive dysfunction** | **Family history of bronchiectasis** |  |
| 44 | M | 19 | Y | Y | N | N | NA | N | Undefined |
| 45 | F | 35 | Y | Y | N | N | Y | Y | Undefined |
| 46 | M | 38 | N | N | N | N | N | Y | Undefined |
| 47 | F | 49 | N | Y | N | N | Y | N | Undefined |
| 48 | F | 29 | Y | Y | N | N | NA | Y | Undefined |
| 49 | M | 30 | N | Y | N | N | N | N | Undefined |
| 50 | M | 15 | Y | Y | N | N | NA | N | Undefined |
| 51 | F | 26 | Y | Y | N | N | NA | N | Undefined |
| 52 | F | 42 | N | N | N | N | N | Y | Undefined |
| 53 | F | 20 | Y | N | N | N | NA | N | Undefined |
| 54 | M | 47 | Y | Y | N | N | N | Y | Undefined |
| 55 | M | 15 | Y | N | N | N | NA | N | Undefined |
| 56 | M | 30 | Y | N | N | N | NA | N | Undefined |
| 57 | F | 16 | Y | Y | Y | N | NA | N | Undefined |
| 58 | F | 53 | Y | N | N | N | N | N | Undefined |
| 59 | F | 13 | Y | Y | Y | N | NA | N | Undefined |
| 60 | F | 20 | Y | Y | N | N | NA | N | Undefined |
| 61 | M | 46 | N | N | N | N | N | Y | Undefined |
| 62 | M | 53 | Y | N | N | N | N | N | Undefined |
| 63 | M | 46 | Y | N | Y | N | Y | N | Undefined |
| 64 | F | 32 | Y | N | N | N | N | N | Undefined |

(Continued)

**e-Table 1** *(Continued)*

| **Patient No.^§^** | **Demographic information** | | **Clinical characteristics** | | | | | | **Diagnosis** |
| --- | --- | --- | --- | --- | --- | --- | --- | --- | --- |
|  | **Gender** | **Age**  **(years)** | **Respiratory symptoms from childhood** | **Rhinosinusitis** | **Otitis media** | **Organ laterality defects** | **Reproductive dysfunction** | **Family history of bronchiectasis** |  |
| 65 | F | 27 | Y | N | N | N | N | N | Undefined |
| 66 | M | 59 | Y | N | N | N | N | N | Undefined |
| 67 | F | 44 | N | Y | N | N | N | N | Undefined |
| 68 | F | 41 | N | Y | N | N | Y | N | Undefined |
| 69 | M | 21 | Y | Y | N | N | Y | N | Undefined |
| 70 | M | 35 | Y | N | N | N | N | N | Undefined |
| 71 | F | 21 | Y | Y | N | N | NA | N | Undefined |
| 72 | F | 33 | Y | N | N | N | N | N | Undefined |
| 73 | F | 25 | Y | Y | N | N | NA | N | Undefined |
| 74 | F | 35 | Y | Y | Y | N | N | N | Undefined |
| 75 | F | 55 | N | N | Y | N | N | Y | Undefined |
| 76 | F | 69 | N | N | N | N | N | Y | Undefined |
| 77 | F | 32 | Y | Y | Y | N | NA | N | Undefined |
| 78 | F | 13 | Y | Y | N | N | NA | N | Undefined |
| 79 | F | 36 | N | Y | N | N | N | N | Undefined |
| 80 | F | 56 | N | N | Y | N | N | Y | Undefined |
| 81 | F | 57 | N | Y | Y | N | N | N | Undefined |
| 82 | F | 14 | Y | Y | N | N | NA | N | Undefined |
| 83 | F | 31 | N | Y | Y | N | NA | N | Undefined |
| 84 | F | 22 | Y | Y | N | N | NA | N | Undefined |
| 85 | F | 37 | Y | Y | Y | N | NA | N | Undefined |

(Continued)

**e-Table 1** *(Continued)*

| **Patient No.^§^** | **Demographic information** | | **Clinical characteristics** | | | | | | **Diagnosis** |
| --- | --- | --- | --- | --- | --- | --- | --- | --- | --- |
|  | **Gender** | **Age**  **(years)** | **Respiratory symptoms from childhood** | **Rhinosinusitis** | **Otitis media** | **Organ laterality defects** | **Reproductive dysfunction** | **Family history of bronchiectasis** |  |
| 86 | F | 29 | Y | Y | Y | N | NA | N | Undefined |
| 87 | F | 22 | N | Y | N | N | NA | N | Undefined |

CF: cystic fibrosis; F: female; M: male; N: no; NA: not available; PCD: primary ciliary dyskinesia; Y: yes.

**^§^** The same numbers in e-Table 1 and e-Table 2 correspond to the same patients.

**e-Table 2** Diagnostic information of all 87 patients

| **Patient No.^§^** | **WES results** | | | | **Sweat chloride value, mmol/L^#^** | **nNO (nl/min)** | **TEM** | **Kartagener syndrome** | **Diagnosis** |
| --- | --- | --- | --- | --- | --- | --- | --- | --- | --- |
|  | **Gene** | **Genotype** | **Mutations** | **ACMG** |  |  |  |  |  |
| 1 | *CFTR* | Compound heterozygous | c.264_268del(p.Leu88Phefs*21) | P | NA | NA | NA | N | CF |
|  |  |  | c.3717G>A(p.Arg1239=) | P |  |  |  |  |  |
| 2 | *CFTR* | Compound heterozygous | c.2909G>A(p.Gly970Asp) | P | 136 | NA | NA | N | CF |
|  |  |  | c.3745G>C(p.Gly1249Arg) | LP |  |  |  |  |  |
| 3 | *CFTR* | Compound heterozygous | c.3068T>G(p.Ile1023Arg) | P | 114 | NA | NA | N | CF |
|  |  |  | c.2909G>A(p.Gly970Asp) | P |  |  |  |  |  |
| 4 | *CFTR* | Compound heterozygous | EX4-EX11 Del | P | 124 | 39.3 | NA | N | CF |
|  |  |  | c.2374C>T(p.Arg792*) | P |  |  |  |  |  |
| 5 | *CFTR* | Compound heterozygous | c.2036G>A(p.Trp679*) | P | NA | NA | NA | N | CF |
|  |  |  | c.164+2T>C | LP |  |  |  |  |  |
| 6 | *CFTR* | Homozygous | EX20 Del | LP | 142 | 10.9 | NA | N | CF |
| 7 | *CFTR* | Compound heterozygous | c.214G>A(p.Ala72Thr) | VUS→LP^¶^ | 34/41 | 310 | NA | N | CF |
|  |  |  | c.3406G>A(p.Ala1136Thr) | VUS→LP^¶^ |  |  |  |  |  |
| 8 | *CFTR* | Homozygous | c.3718-2477C>T | P | 70 | 152.7 | NA | N | CF |
| 9 | CFTR | Compound heterozygous | c.595C>T(p.His199Tyr) | P | 108 | NA | NA | N | CF |
|  |  |  | c.2060_2061del(p.Phe687*) | LP |  |  |  |  |  |
| 10 | CFTR | Compound heterozygous | c.3140-26A>G | P | 124 | NA | NA | N | CF |
|  |  |  | EX2-EX3 Del | P |  |  |  |  |  |
| 11 | CFTR | Compound heterozygous | c.580-1G>T | P | 135 | 54.5 | NA | N | CF |
|  |  |  | c.1000C>T(p.Arg334Trp) | P |  |  |  |  |  |
| 12 | CFTR | Compound heterozygous | c.1657C>T(p.Arg553*) | P | 116 | NA | NA | N | CF |
|  |  |  | c.869+5G>A | LP |  |  |  |  |  |
| 13 | CFTR | Compound heterozygous | c.2909G>A(p.Gly970Asp) | P | NA | NA | NA | N | CF |
|  |  |  | EX6-EX7 Del | LP |  |  |  |  |  |
| (Continued) | | | | | | | | | |

**e-Table 2** *(Continued)*

| **Patient No.^§^** | **WES results** | | | | **Sweat chloride value, mmol/L^#^** | **nNO (nl/min)** | **TEM** | **Kartagener syndrome** | **Diagnosis** |
| --- | --- | --- | --- | --- | --- | --- | --- | --- | --- |
|  | **Gene** | **Genotype** | **Mutations** | **ACMG** |  |  |  |  |  |
| 14 | *CFTR* | Homozygous | c.2909G>A(p.Gly970Asp) | P | 69 | 81.5 | NA | N | CF |
| 15 | *CFTR* | Compound heterozygous | c.3909C>G(p.Asn1303Lys) | P | NA | NA | NA | N | CF |
|  |  |  | c.1699G>T(p.Asp567Tyr) | LP |  |  |  |  |  |
| 16※ | *CCDC40* | Heterozygous | c.961C>T(p.Arg321*) | P | NA | 7.2 | NA | N | PCD |
| 17 | *CCNO* | Homozygous | c.425delC(p.Pro142Argfs*15) | P | NA | NA | NA | N | PCD |
| 18 | *CFAP300* | Homozygous | c.110+1G>A | P | NA | 5.2 | NA | N | PCD |
| 19 | *DNAAF1* | Compound heterozygous | c.376del(p.Glu126Lysfs*35) | P | NA | 11.0 | NA | N | PCD |
|  |  |  | c.394del(p.Arg132Alafs*29) | LP |  |  |  |  |  |
| 20 | *DNAAF11* | Homozygous | c.290_291del(pVal97Glufs*10) | LP | NA | 3.0 | NA | Y | PCD |
| 21 | *DNAAF11* | Homozygous | c.1020C>G(p.Tyr340*) | LP | NA | 5.0 | NA | Y | PCD |
| 22 | *DNAAF4* | Homozygous | c.30G>A (p.Trp10*) | LP | NA | 16.3 | NA | N | PCD |
| 23 | *DNAAF6** | Hemizygous | c.532_533delCT(p.Leu178Serfs*2) | LP | NA | 6.2 | NA | N | PCD |
| 24 | *DNAH11* | Homozygous | EX21-EX66 Del | LP | NA | 3.3 | NA | N | PCD |
| 25 | *DNAH11* | Compound heterozygous | c.6727C>T(p.Arg2243*) | P | NA | NA | NA | Y | PCD |
|  |  |  | c.11104C>T(p.Arg3702*) | LP |  |  |  |  |  |
| 26 | *DNAH11* | Homozygous | c.13176_13178delCTT(p.Phe4392del) | LP | NA | 20.0 | IDA | N | PCD |
| 27 | *DNAH11* | Compound heterozygous | c.6685C>T(p.Arg2229*) | P | NA | 8.3 | NA | N | PCD |
|  |  |  | c.8554C>T(p.Arg2852*) | P |  |  |  |  |  |
| 28 | *DNAH5* | Compound heterozygous | c.9502C>T(p.Arg3168*) | P | <30 | 3.7 | NA | N | PCD |
|  |  |  | c.3441_3442del(p.Lys1114Argfs*10) | LP |  |  |  |  |  |
| 29 | *DNAH5* | Compound heterozygous | c.8597delG(p.Gly2866Glufs*10） | P | NA | 7.1 | NA | Y | PCD |
|  |  |  | EX79 Del | LP |  |  |  |  |  |
| 30 | *DNAH5* | Compound heterozygous | c.9213del(p.His3071Glnfs*5) | LP | NA | 5.8 | NA | N | PCD |
|  |  |  | c.6786del(p.Ser2264Valfs*12) | LP |  |  |  |  |  |
| (Continued) | | | | | | | | | |

**e-Table 2** *(Continued)*

| **Patient No.^§^** | **WES results** | | | | **Sweat chloride value, mmol/L^#^** | **nNO (nl/min)** | **TEM** | **Kartagener syndrome** | **Diagnosis** |
| --- | --- | --- | --- | --- | --- | --- | --- | --- | --- |
|  | **Gene** | **Genotype** | **Mutations** | **ACMG** |  |  |  |  |  |
| 31 | *DNAH5* | Compound heterozygous | c.9235C>T(p.Arg3079*) | LP | NA | 19.1 | NA | Y | PCD |
|  |  |  | c.5563dup(p.Ile1855Asnfs*6) | P |  |  |  |  |  |
| 32 | *DNAH5* | Compound heterozygous | c.7504_7509delCGCCGCinsAGCTGGAG  (p.Arg2502Serfs*70) | P | NA | 3.6 | NA | Y | PCD |
|  |  |  | c.8821-1G>T | LP |  |  |  |  |  |
| 33 | *ODAD1* | Compound heterozygous | c.71-2A>C | LP | NA | 6.7 | NA | Y | PCD |
|  |  |  | c.813_816dup(p.Pro273Alafs*11) | P |  |  |  |  |  |
| 34 | *ODAD4* | Compound heterozygous | c.246+1G>T | VUS | NA | 314.8 | NA | Y | PCD |
|  |  |  | c.1528+1G>T | LP |  |  |  |  |  |
| 35 | *OFD1** | Hemizygous | c.2843_2844delAA(p.Lys948Argfs*7) | P | NA | 127.7 | NA | N | PCD |
| 36 | *RSPH3* | Compound heterozygous | c.1105C>T(p.Arg369*) | P | NA | 45.6 | NA | N | PCD |
|  |  |  | c.1096C>T(p.Gln366*) | LP |  |  |  |  |  |
| 37 | *RSPH4A* | Compound heterozygous | c.1889_1895dup(p.Tyr633Trpfs*12) | LP | NA | 6.8 | CA/MTD | N | PCD |
|  |  |  | c.2T>C(p.Met1?) | VUS |  |  |  |  |  |
| 38 | *RSPH4A* | Homozygous | c.1391G>A(p.Gly464Glu) | P | NA | 30.6 | NA | N | PCD |
| 39 | *RSPH4A* | Compound heterozygous | c.1475C>T(p.Ser492Leu) | VUS | NA | 28.8 | CA | N | PCD |
|  |  |  | c.1764G>T(p.Gly588=) | VUS |  |  |  |  |  |
| 40 | *RSPH9* | Homozygous | c.574G>T(p.Glu192*) | LP | NA | 5.2 | NA | N | PCD |
| 41※ | Negative | / | / | / | NA | 8.2 | NA | N | PCD |
| 42 | Novel gene^¶^ | Compound heterozygous | / | P | NA | 7.0 | NA | N | PCD |
| 43 | *GATA2** | Heterozygous | c.1099_1105del(p.Asp367Serfs*18) | LP | NA | NA | NA | N | Immunodeficiency-21 |

(Continued)

**e-Table 2** *(Continued)*

| **Patient No.^§^** | **WES results** | | | | **Sweat chloride value, mmol/L^#^** | **nNO (nl/min)** | **TEM** | **Kartagener syndrome** | **Diagnosis** |
| --- | --- | --- | --- | --- | --- | --- | --- | --- | --- |
|  | **Gene** | **Genotype** | **Mutations** | **ACMG** |  |  |  |  |  |
| 44 | *CFTR* | Heterozygous | c.1210-11T>G | P | NA | NA | NA | N | Undefined |
| 45 | Negative | / | / | / | NA | NA | NA | N | Undefined |
| 46 | *STAT3** | Heterozygous | c.92G>A(p.Arg31Gln) | VUS | < low limit of detection | NA | NA | N | Undefined |
| 47 | Negative | / | / | / | NA | 359.6 | NA | N | Undefined |
| 48 | *HYDIN* | Compound heterozygous | c.1670+1G>C | LP | NA | 4.2 | NA | N | Undefined |
|  |  |  | c.9774+5G>C | VUS |  |  |  |  |  |
| 49 | *DRC1* | Heterozygous | c.1509+12C>T | VUS | NA | 172.5 | NA | N | Undefined |
| 50 | *CFTR* | Heterozygous | c.3468G>T(p.Leu1156Phe) | VUS | < low limit of detection | 422.1 | NA | N | Undefined |
| 51 | Negative | / | / | / | NA | 335.7 | NA | N | Undefined |
| 52 | Negative | / | / | / | NA | 326.4 | NA | N | Undefined |
| 53 | Negative | / | / | / | NA | NA | NA | N | Undefined |
| 54 | Negative | / | / | / | NA | NA | NA | N | Undefined |
| 55 | *CFTR* | / | (TG)13(T)5/(TG)11(T)7 | / | NA | NA | NA | N | Undefined |
| 56 | *RSPH4A* | Heterozygous | c.1774_1775delTT(p.Leu592Aspfs*5) | P | < low limit of detection | NA | NA | N | Undefined |
| 57 | Negative | / | / | / | NA | 180.6 | NA | N | Undefined |
| 58 | *TNFRSF13B** | Heterozygous | c.704_705delCT(p.Pro235Argfs*169) | VUS | NA | 383.9 | NA | N | Undefined |
| 59 | *DNAH11* | Heterozygous | c.2749G>T(p.Glu917*) | LP | NA | 297.4 | NA | N | Undefined |
| 60 | *DNAH1* | Heterozygous | c.11726_11727delCT(p.Pro3909Argfs*33) | P | NA | 24.4 | NA | N | Undefined |

(Continued)

**e-Table 2** *(Continued)*

| **Patient No.^§^** | **WES results** | | | | **Sweat chloride value, mmol/L^#^** | **nNO (nl/min)** | **TEM** | **Kartagener syndrome** | **Diagnosis** |
| --- | --- | --- | --- | --- | --- | --- | --- | --- | --- |
|  | **Gene** | **Genotype** | **Mutations** | **ACMG** |  |  |  |  |  |
| 61 | Negative | / | / | / | < low limit of detection | 276.1 | NA | N | Undefined |
| 62 | Negative | / | / | / | NA | 264.9 | NA | N | Undefined |
| 63 | Negative | / | / | / | < low limit of detection | 193.9 | NA | N | Undefined |
| 64 | Negative | / | / | / | NA | 287.4 | NA | N | Undefined |
| 65 | Negative | / | / | / | NA | 181.4 | NA | N | Undefined |
| 66 | Negative | / | / | / | NA | 14.4 | NA | N | Undefined |
| 67 | Negative | / | / | / | NA | 34.6 | NA | N | Undefined |
| 68 | *IL6ST** | Heterozygous | c.1115G>A(p.Arg372Lys) | VUS | NA | 146.7 | NA | N | Undefined |
| 69 | *CFTR* | Heterozygous | c.1069G>A(p.Ala357Thr) | VUS | NA | 196.7 | NA | N | Undefined |
| 70 | *NFKB2** | Heterozygous | c.797A>G(p.Asp266Gly) | VUS | 34/40 | NA | NA | N | Undefined |
| 71 | *DNAH1* | Compound heterozygous | c.7429C>T(p.Arg2477Trp) | VUS | NA | 244.8 | NA | N | Undefined |
|  |  |  | c.9353G>A(p.Arg3118Gln) | VUS |  |  |  |  |  |
| 72 | Negative | / | / | / | NA | 101.8 | NA | N | Undefined |
| 73 | *SCNN1G** | Heterozygous | c.773T>C(p.Val258Ala) | VUS | NA | 91.3 | NA | N | Undefined |
| 74 | *DNAH11* | Heterozygous | c.3025C>G(p.Leu1009Val) | VUS | NA | 81.2 | NA | N | Undefined |
| 75 | Negative | / | / | / | NA | 197.6 | NA | N | Undefined |
| 76 | *DNAH1* | Compound heterozygous | c.6060G>T(p.Glu2020Asp) | VUS | NA | 172.9 | NA | N | Undefined |
|  |  |  | c.8986C>T(p.Leu2996Phe) | VUS |  |  |  |  |  |
| 77 | *CFTR* | Compound heterozygous | 7q31.2 Del | P | < low limit of detection | 233.4 | NA | N | Undefined |
|  |  |  | c.2991G>C(p.Leu997Phe) | VUS |  |  |  |  |  |

(Continued)

**e-Table 2** *(Continued)*

| **Patient No.^§^** | **WES results** | | | | **Sweat chloride value, mmol/L^#^** | **nNO (nl/min)** | **TEM** | **Kartagener syndrome** | **Diagnosis** |
| --- | --- | --- | --- | --- | --- | --- | --- | --- | --- |
|  | **Gene** | **Genotype** | **Mutations** | **ACMG** |  |  |  |  |  |
| 78 | Negative | / | / | / | NA | 328.2 | Inadequate specimen | N | Undefined |
| 79 | Negative | / | / | / | NA | NA | NA | N | Undefined |
| 80 | Negative | / | / | / | NA | 183.8 | NA | N | Undefined |
| 81 | Negative | / | / | / | NA | 424.4 | NA | N | Undefined |
| 82 | *PIK3CD** | Heterozygous | c.19T>C (p.Cys7Arg) | VUS | NA | 221.1 | NA | N | Undefined |
| 83 | Negative | / | / | / | NA | 40.6 | NA | N | Undefined |
| 84 | Negative | / | / | / | NA | NA | NA | N | Undefined |
| 85 | Negative | / | / | / | NA | 47.1 | NA | N | Undefined |
| 86 | Negative | / | / | / | NA | NA | NA | N | Undefined |
| 87 | Negative | / | / | / | NA | 66.2 | NA | N | Undefined |

ACMG: American College of Medical Genetics and Genomics; CA: central apparatus defects; CF: cystic fibrosis; IDA: inner dynein arm defects; LP: likely pathogenic; MTD: microtubule disorganization; N: no; NA: not available; nNO: nasal nitric oxide measurement; P: pathogenic; PCD: primary ciliary dyskinesia; VUS: variant of uncertain significance; WES: whole exome sequencing; Y: yes.

**^§^** The same numbers in e-Table 1 and e-Table 2 correspond to the same patients.

^#^ Each patient undergoes two sweat chloride tests. If the results are both greater than 60mmol/L, record the low value. Otherwise, record the results of two separate tests.

^¶^ Re-analyze the raw genetic testing data.

※The patient has a low nNO level and at least two key clinical features for PCD, and subsequent WES did not detect *CFTR* mutations, so they can be clinically diagnosed with PCD.

* *DNAAF6* and *OFD1* are X-linked inheritance, *GATA2*, *STAT3, IL6ST, NFKB2, SCNN1G and PIK3CD* are autosomal dominant inheritance, *TNFRSF13B* is autosomal dominant or autosomal recessive inheritance, and all other genes are autosomal recessive inheritance.
